# Childhood community-acquired pneumonia

**DOI:** 10.1007/s00431-023-05366-6

**Published:** 2023-12-19

**Authors:** Patrick M. Meyer Sauteur

**Affiliations:** Division of Infectious Diseases and Hospital Epidemiology, University Children’s Hospital Zurich, Steinwiesstrasse 75, CH-8032 Zurich, Switzerland

**Keywords:** Antibiotics, Antimicrobial resistance, Colonisation, Diagnosis, Respiratory tract infection, Vaccine

## Abstract

Community-acquired pneumonia (CAP) is a common disease in children, and its aetiological and clinical diagnosis are challenging for physicians in both private practice and hospitals. Over the past three decades, conjugate vaccines have successfully reduced the burden of the former main causes of CAP, *Streptococcus pneumoniae* and *Haemophilus influenzae* type b. Today, viruses are by far the most commonly detected pathogens in children with CAP.

*  Conclusion*: New insights into the aetiology and treatment of CAP in children in recent years have influenced management and are the focus of this review. In addition to reducing diagnostic uncertainty, there is an urgent need to reduce antibiotic overuse and antimicrobial resistance in children with CAP.

**Table:** 

**What is Known:** *• Conjugate vaccines against Streptococcus pneumoniae and Haemophilus influenzae type b have shifted the epidemiology of childhood CAP to predominantly viral pathogens and Mycoplasma pneumoniae.* *• Clinical, laboratory, and radiological criteria cannot reliably distinguish between bacterial and viral aetiology in children with CAP.*	
**What is New:** *• Test results and epidemiological data must be carefully interpreted, as no single diagnostic method applied to non-pulmonary specimens has both high sensitivity and high specificity for determining pneumonia aetiology in childhood CAP.* *• This review provides a simple and pragmatic management algorithm for children with CAP to aid physicians in providing optimal and safe care and reducing antibiotic prescribing.*

**What is Known:**

*• Conjugate vaccines against Streptococcus pneumoniae and Haemophilus influenzae type b have shifted the epidemiology of childhood CAP to predominantly viral pathogens and Mycoplasma pneumoniae.*

*• Clinical, laboratory, and radiological criteria cannot reliably distinguish between bacterial and viral aetiology in children with CAP.*

**What is New:**

*• Test results and epidemiological data must be carefully interpreted, as no single diagnostic method applied to non-pulmonary specimens has both high sensitivity and high specificity for determining pneumonia aetiology in childhood CAP.*

*• This review provides a simple and pragmatic management algorithm for children with CAP to aid physicians in providing optimal and safe care and reducing antibiotic prescribing.*

## Introduction

Community-acquired pneumonia (CAP) is an acute infection of the lung parenchyma acquired outside the hospital or other health care settings. It is one of the most common causes of hospitalization in children in developed countries [1] and the leading cause of death in children in developing countries [2, 3]. Clinical diagnosis of CAP is difficult because symptoms vary with age and may be nonspecific in young children. In addition, determining the aetiology of CAP remains a major challenge. The last guidelines for CAP in children from the Infectious Diseases Society of America (IDSA) [4] and the British Thoracic Society (BTS) [5] were published more than a decade ago. During this time period, new insights into the aetiology and treatment of childhood CAP have influenced management and are the focus of this review.

## Epidemiology

**Fig. 1:**
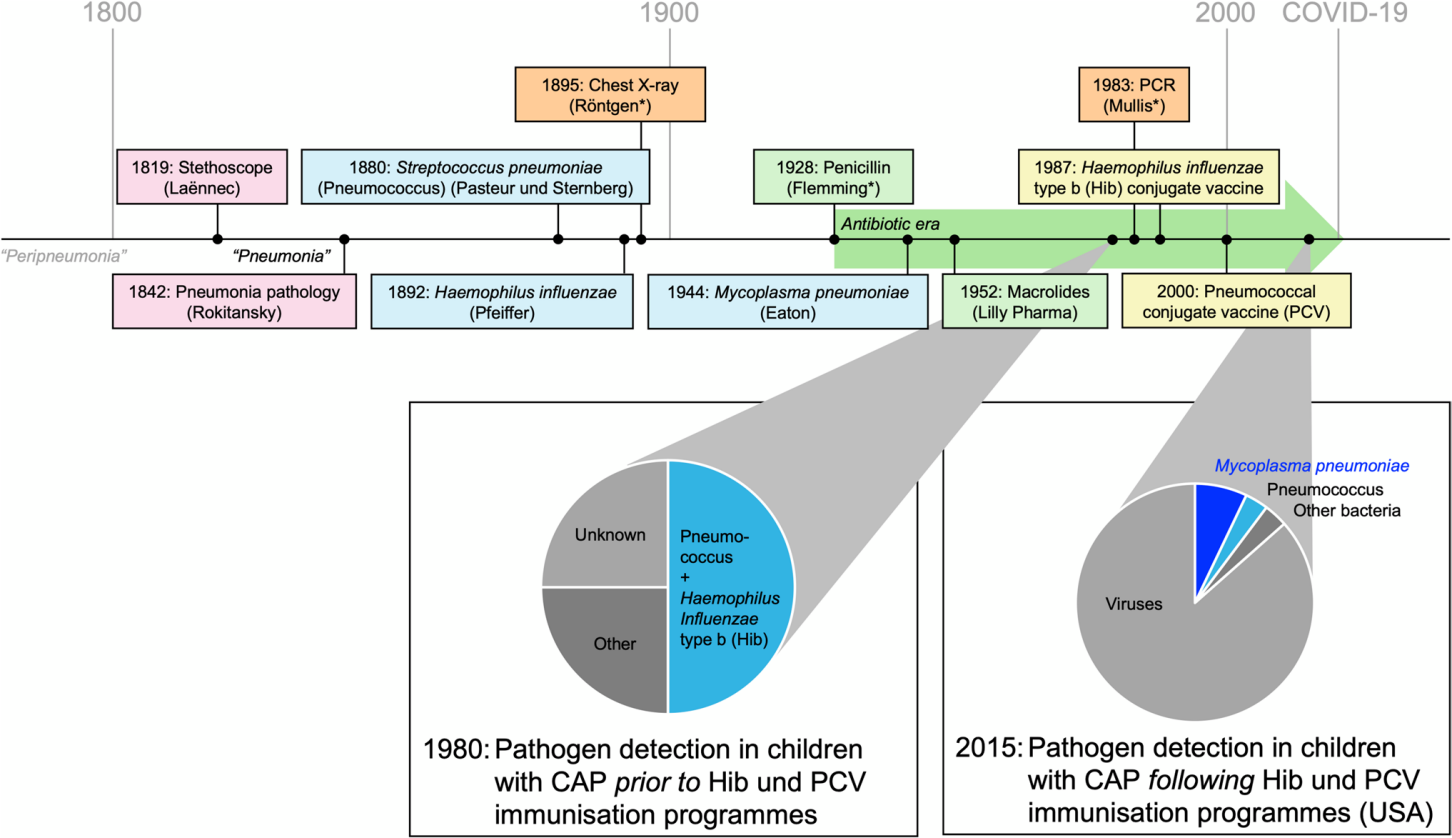
Milestones and changes in the aetiology of childhood pneumonia. Abbreviations: Hib, *Haemophilus influenzae* type b; PCR, polymerase chain reaction; PCV, pneumococcal conjugate vaccine. Pie charts adapted from Feikin et al. [3] and Jain et al. [1]. Surnames of inventors/discoverers are shown in parentheses. *Nobel laureates. The history of defined pneumonia dates back only to 1800. “Peripneumonia” was used prior to that to describe a clinical pattern with no distinction between pneumonia and pleuritis. The first milestones included a precise clinical description with the invention of the stethoscope (auscultatory findings) and autopsy (differentiation between lobar pneumonia and bronchopneumonia). This was followed by other milestones, such as the first description of pneumonia-causing pathogens (*Streptococcus pneumoniae*, *Haemophilus influenzae*, and *Mycoplasma pneumoniae*), the invention of X-ray technology, and initiation of the antibiotic era with the discovery of penicillin. The invention of PCR allowed the detection of several bacterial and viral pathogens, the distribution of which was significantly influenced by the development and introduction of conjugate vaccines against *H. influenzae* type b (Hib) and *S. pneumoniae* (PCV)

The incidence and aetiological spectrum of CAP have changed substantially with the introduction of conjugate vaccines against the former main causes of CAP, *Haemophilus influenzae* type b (Hib) and *Streptococcus pneumoniae* (pneumococcus) (Fig. 1). Hib immunization programmes have reduced CAP rates in low- and high-income settings [5]. The later implementation of pneumococcal conjugate vaccine (PCV) resulted in a reduction in invasive pneumococcal disease (IPD) as well as a further reduction in CAP incidence and admission rates in both settings [4]. The extended valency of PCV from 7-valent (PCV7) to 13-valent (PCV13) has also led to reduced infections with resistant pneumococcal strains due to the inclusion of non-susceptible *S. pneumoniae* serotypes, mainly serotype 19A [6].

Recent large-scale studies have performed extensive microbiological testing to investigate the aetiology in children with radiologically confirmed CAP. A viral and/or bacterial pathogen was detected in the upper respiratory tract (URT) in 81–99% of these children. Viruses accounted for the majority of pathogens [1–3], particularly in young children (> 90%) [2].

Prior to the COVID-19 pandemic, the most common pathogen detected in hospitalized children with CAP in the USA was respiratory syncytial virus (RSV) [1]. The most commonly detected bacterial pathogen was *Mycoplasma pneumoniae* [1]. However, the detection of pathogens varied with age (Table 1). The proportion of RSV was significantly higher in children < 5 years of age compared with older children (37% vs. 8%). In contrast, the proportion of *M. pneumoniae* was higher in children ≥ 5 years of age compared with younger children (19% vs. 3%) [1].

**Table 1:** Pathogens detected in children with CAP according to age group

**Age**	** < 5 years**	** ≥ 5 years**
Pathogens^a^	Respiratory viruses (predominantly RSV) *Streptococcus pneumoniae*	Respiratory viruses *Mycoplasma pneumoniae* *Streptococcus pneumoniae*

*RSV*, respiratory syncytial virus. Table adapted from Haq et al. [6]

^a^According to frequency

The introduction of nonpharmaceutical interventions (NPIs) against COVID-19 in early 2020 resulted in the disappearance of almost all respiratory pathogens. Interestingly, reductions in pneumococcal CAP and IPD were not predominantly related to reduced pneumococcal carriage and density but were associated with the disappearance of respiratory viruses such as RSV, influenza viruses, and human metapneumovirus [7]. The circulation of SARS-CoV-2 had little impact on the incidence of CAP, as COVID-19 did not primarily manifest as CAP in immunocompetent children. The lifting of NPIs in 2021 has led to a resurgence of most respiratory pathogens. The re-emergence of *M. pneumoniae* was delayed until autumn 2023 [8].

## Diagnosis

### Clinical diagnosis

Childhood CAP is mainly diagnosed clinically, but symptoms and signs vary with age and are highly variable. CAP should be considered in children with fever and tachypnoea after reducing fever with antipyretics [5]. Apart from tachypnoea, additional signs of respiratory distress in children with CAP can include chest indrawing (suprasternal, intercostal, or subcostal), nasal flaring, and grunting [4]. Other indicative clinical symptoms and signs include cough, chest or abdominal pain, and focal chest sign(s). Tachypnoea appears to be the most important clinical sign because it correlates with hypoxemia, pulmonary infiltrates on chest radiograph, and the overall severity of CAP [5]. The condition is defined according to age-related reference values: < 2 months, > 60 breaths/min; 2–12 months, > 50/min; 1–5 years, > 40/min; and > 5 years, > 20/min [4]. The respiratory rate should be counted for 1 min when the child is quiet. Fever alone can increase the respiratory rate by 10 breaths/min/°C of body temperature.

Chest radiography correlates poorly with clinical signs and outcome and should therefore not be considered as a routine investigation [4, 5]. However, lung ultrasonography with its portability, safety, and wide availability may be a useful screening tool, also to exclude CAP in patients who would most likely benefit from only clinical observation and symptomatic treatment [9].

Numerous studies have demonstrated that clinical, laboratory, and radiological criteria cannot reliably distinguish between bacterial and viral aetiology in children with CAP [4, 5]. Biomarkers such as C-reactive protein (CRP) or serum procalcitonin (PCT) are not useful to differentiate viral and bacterial causes of CAP, but can guide investigation and management of complicated CAP in children as defined below [6]. Therefore, treatment decisions should be based on the expected pathogens according to the epidemiology and age of the child.

### Microbiological diagnosis

**Fig. 2:**
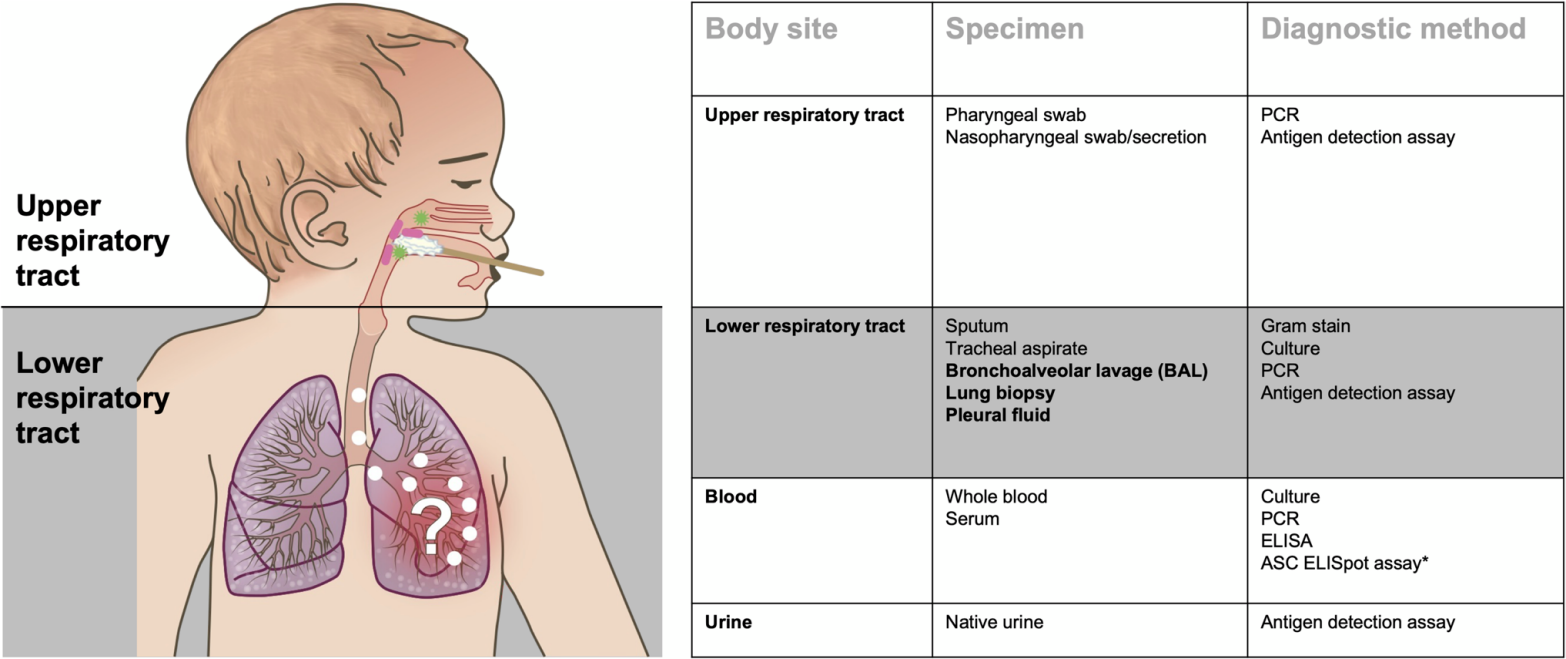
Specimens and diagnostic methods for the microbiological diagnosis of CAP in children. Abbreviations: ASC, antibody-secreting cell; ELISA, enzyme-linked immunosorbent assay (serology); ELISpot, enzyme-linked immunospot assay (cell-based assay); PCR, polymerase chain reaction. Figure adapted from Meyer Sauteur [10]. Samples taken directly from the lungs are shown in bold and are the “gold standard” for the microbiological diagnosis of CAP. *The detection of pathogen-specific ASCs by ELISpot is not yet a validated method for the microbiological diagnosis of CAP

Timely and reliable identification of the underlying pathogen is critical for initiating effective and tailored antimicrobial treatment, but identifying the microbial aetiology of pneumonia is challenging in many clinical settings [10]. Microbiological testing is generally recommended to attempt an aetiological diagnosis of CAP patients requiring hospitalization [4, 5]. The “gold standard” for determining pneumonia aetiology is the detection of respiratory pathogens in specimens taken directly from the lungs by bronchoalveolar lavage, pleural fluid sampling, or lung biopsy or aspiration (Fig. 2). Because these methods are invasive and require anaesthesia in children, they are rarely performed in clinical practice.

Sputum and tracheal aspirates are samples from the lower respiratory tract with a higher probability of URT contamination. In addition, sputum collection is hampered in children by difficulties with expectoration. Therefore, the aetiological diagnosis of CAP mostly depends on the detection of respiratory pathogens from specimens distant from the site of infection, such as URT samples, blood, and urine. However, test results from these specimens must be carefully interpreted because no single diagnostic method applied to these non-pulmonary specimens has both high sensitivity and high specificity for determining CAP aetiology [10]. For example, in a large multi-country case–control study, multiplex PCR detected four or more pathogens in the URT of more than half of childhood pneumonia cases (59%) and healthy controls (54%) [3]. Only RSV was rarely detected in the URT of healthy controls [2, 3]. Overall, the detection of several potential pathogens in the URT of children with CAP may represent carriage, asymptomatic infection, URT infection without lower respiratory tract involvement, or persistence after infection. This complicates the assignment of causative pathogens for a particular CAP episode. Blood cultures are not sensitive because they are only positive in approximately 2% of hospitalized children with CAP [4]. Pneumococcal urine antigen tests exhibit poor specificity and are also positive in patients who carry *S. pneumoniae* in the URT [5]. *Streptococcus pneumoniae* can be detected in the URT of up to 77% and 34% of healthy children and adults, respectively [10]. In addition, carriage elicits systemic antibody responses, limiting serology as a diagnostic test to reliably determine pneumonia aetiology.

Promising diagnostic approaches for the future are novel biomarkers, exhaled-breath analysis, and multidimensional molecular assessment of the host response [10], as well as new analytical approaches [3]. In CAP caused by *M. pneumoniae*, we demonstrated that the detection of pathogen-specific antibody-secreting cells (ASCs) by enzyme-linked immunospot (ELISpot) assay improved the diagnosis of *M. pneumoniae* infection [11]. *Mycoplasma pneumoniae*–specific IgM ASCs were detectable in children with *M. pneumoniae* CAP but not in *M. pneumoniae* carriers suffering from CAP caused by other pathogens or asymptomatic *M. pneumoniae* carriers [12]. This method is now being validated and extended to other CAP pathogens.

## Treatment

### Management

**Fig. 3:**
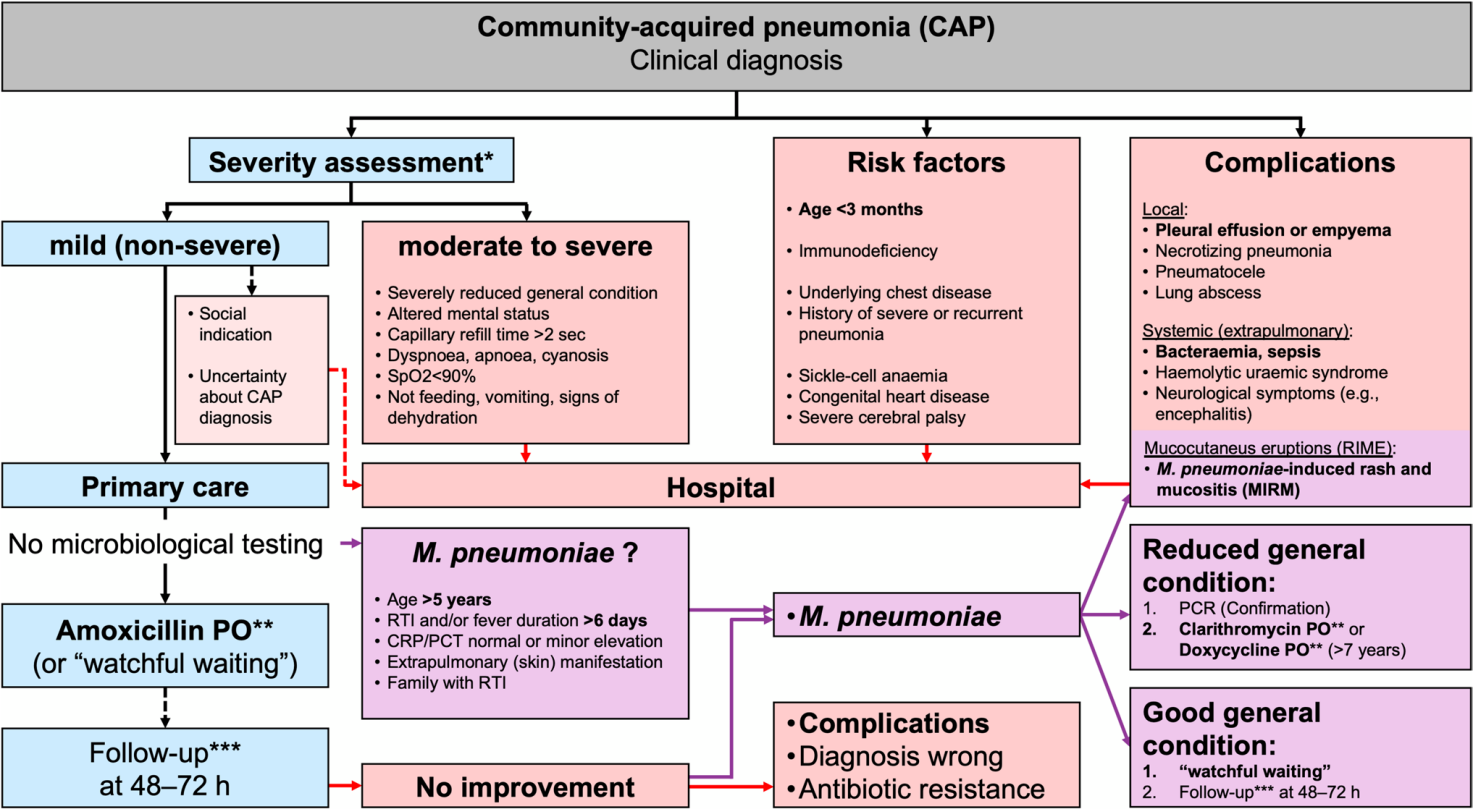
Algorithm for the management of CAP in children. Abbreviations: CRP, C-reactive protein; MIRM, *M. pneumoniae*–induced rash and mucositis; PCR, polymerase chain reaction; PCT, procalcitonin; PO, oral treatment; RIME, reactive infectious mucocutaneous eruption; RTI, respiratory tract infection; SpO_2_, saturation of peripheral oxygen. Figure adapted from Haq et al. [6]. *No specific score is available to assess the severity of CAP in children. **Details on dosage and duration of antibiotic treatment are given in Table 2. ***Parents are advised that they should call for a follow-up appointment at 48–72 h in case of non-response to empirical treatment. No follow-up is required if the child has already improved previously. In case of clinical deterioration, immediate presentation is required

Because of the diagnostic uncertainty, children with CAP are often prescribed antibiotics “just in case” for fear of rapid clinical deterioration, future hospital admission, or complications of bacterial infection. Across health care systems, antibiotic prescription increases with diagnostic uncertainty [13]. CAP is a major reason for prescribing antibiotics in children. The vast majority of these infections are managed in primary care, where 80% of all prescriptions for antibiotics are obtained and where the use of antibiotics has been shown to directly affect the development of antimicrobial resistance (AMR) [13]. Reducing diagnostic uncertainty by identifying children with CAP who are at risk of bacterial infection and ensuing complications could significantly reduce inappropriate antibiotic prescription and use. A risk assessment may support physicians in identifying children with CAP at risk for severe disease progression (Fig. 3). Hospital admission is recommended for moderate to severe CAP, the presence of risk factors, or evidence of complications [4, 5]. However, most children with CAP can be managed as outpatients.

### “Watchful waiting”

As the majority of CAP in children is viral, not every patient with non-severe CAP and without risk factors needs to be treated with antibiotics [13]. In such situations, it is possible to withhold antibiotics and to watch and wait (“watchful waiting”). This approach will also help reduce side effects, costs, and the development of AMR.

However, the “watchful waiting” approach should only be used provided that the patient can be followed closely, and given advice about alarming symptoms (i.e., criteria for “moderate to severe” disease, Fig. 3) and when and how to seek further help when the child’s condition fails to improve or deteriorate (“safety-netting”). It is not an option if a lack of compliance is suspected or there are language barriers. Extra precautions should be taken when withholding or delaying antibiotics in CAP patients with comorbidities.

### Antibiotics

Oral amoxicillin is globally the most commonly recommended first-line treatment because it is still effective against the majority of bacterial pathogens that cause CAP, is well tolerated, and inexpensive [4, 5]. In case of penicillin allergy or infections with *M. pneumoniae* or *Chlamydia pneumoniae*, macrolides and tetracyclines can be used at any age or > 7 years of age, respectively, according to the IDSA [4] (Table 2).

**Table 2:** Antibiotic treatment for children with non-severe CAP

**Indication**^a^	**Substance**	**Dosage**^b^	**Duration**
First line	Amoxicillin PO	(25–)^c^ 40–45 mg/kg/dose twice a day (maximum 3000 mg/day)	(3–)^c^ 5 days
Penicillin allergy^d^ or *Mycoplasma pneumoniae* and *Chlamydia pneumoniae*	Clarithromycin PO^e^	7.5 mg/kg/dose twice a day (maximum 1000 mg/day)	5 days
	Doxycycline PO^f^ (> 7 years)	First day: 2 mg/kg/dose twice a day (maximum 200 mg/day) Days 2 to 5: 2 mg/kg/dose once a day (maximum 100 mg/day)	5 days

PO, oral treatment. Table adapted from [4, 5]. In case of moderate to severe CAP, presence of risk factors, or evidence of complications (Fig. 3) and/or if microbiological test results are available, antibiotic choice, dosage, and duration must be reconsidered in this context [4, 5]. Intravenous antibiotics are indicated in children who cannot tolerate oral medicines (e.g., because of vomiting) or have bacteraemia or pulmonary complications (Table 3). In patients receiving intravenous antibiotics, switching to oral antibiotics should be considered if there is clear evidence of improvement based on clinical judgement [5]

^a^As the majority of CAP in children is viral, not every patient with non-severe CAP and with an absence of risk factors needs to be treated with antibiotics [13]

^b^Dosage recommendations according to the Swiss Database for dosing medicinal products in paediatrics (https://db.swisspeddose.ch)

^c^According to results of the CAP-IT study [16]

^d^Patients with *suspected* allergy to penicillins should be evaluated by allergy specialists

^e^Clarithromycin should be preferred to azithromycin because azithromycin promotes the development of AMR due to its very long half-life (48 to 108 h) and the associated long-lasting plasma levels (measurable plasma levels > 1 μg/L up to 30 days following 3-day treatment)

^f^Doxycycline may cause photosensitive skin reactions following visible and UV light exposure. Age restriction according to the IDSA [4]

Several studies have recently investigated different durations and doses of amoxicillin for children with CAP in the outpatient setting. Most national guidelines in both low- and high-income countries recommend durations for 5–10 days, but these recommendations are based on sparse evidence [14]. Because current diagnostic methods cannot reliably distinguish between bacterial and viral CAP, no microbiological testing was performed in most studies for patient enrolment. Consequently, the effect of antibiotics on viral CAP was also evaluated and, therefore, likely underestimated in relation to bacterial CAP, which was the intended target of these studies (i.e., “Pollyanna phenomenon”) [13]. For example, the SAFER study (Canada, 2 centres, 281 children) confirmed that 5 days of amoxicillin was comparable to 10 days in children with radiologically confirmed CAP [15]. However, viruses (predominantly RSV) were detected in about two-thirds of patients in that study who were additionally tested by PCR from nasopharyngeal swabs [15]. The CAP-IT study (UK, 29 centres, 824 children) showed that even 3 days of amoxicillin was non-inferior to 7 days with regard to the need for antibiotic re-treatment [16]. Furthermore, lower doses of amoxicillin (30–50 mg/kg/day) were non-inferior to higher doses (70–90 mg/kg/day) for both treatment durations. However, CAP was exclusively clinically diagnosed in that study (no chest radiography and no microbiological testing). Very young children were predominantly included (median age 2.5 years), so it is likely that the majority of children in this study had viral CAP, which makes it difficult to judge the study result of similar treatment failure with varying doses and duration of amoxicillin [16]. Nevertheless, the clinical diagnosis of CAP in this study reflects real-word practice and is in line with current guidelines; thus, the results may be translated to children with non-severe CAP in the outpatient setting. This is also supported by recent systematic reviews and meta-analyses showing that a short duration of 3–5 days seems equally effective and safe compared with the longer duration of 7–10 days [14, 17].

Current recommendations based on these studies include a treatment duration of 5 days for non-severe CAP in children. If the child has already recovered previously, 3 days may also be appropriate.

### Follow-up

No follow-up is required if the child has already improved with antibiotics or “watchful waiting”. Scheduled follow-ups can be considered based on individual patient conditions or at the request of parents. Parents are typically advised that they should call for a follow-up appointment at 48–72 h in case of non-response to empirical treatment. In case of clinical deterioration, immediate presentation is required (“safety-netting”).

### Non-response to empirical treatment

The possible reasons for a non-response to empirical treatment are diverse and include incorrect diagnosis, antibiotic resistance, or complications of CAP. However, only a small proportion of children with CAP develop complications (Table 3). Most children with CAP improve without sequelae.

**Table 3:** Complications of CAP in children

**Site**	**Complication**	**Pathogens**^a^
Local	Pleural effusion or empyema (~ 1%)	*Streptococcus pneumoniae* *Streptococcus pyogenes* *Staphylococcus aureus*
	Necrotizing pneumonia,^b^	*Streptococcus pneumoniae* *Staphylococcus aureus*
	Pneumatocele^b^	
	Lung abscess^b^	*Staphylococcus aureus* Anaerobes
Systemic (extra-pulmonary)	Bacteraemia, sepsis (~ 1%)^c^	*Streptococcus pneumoniae* *Streptococcus pyogenes* *Staphylococcus aureus*
	Rash, urticaria, mucositis (MIRM)^d^	*Mycoplasma pneumoniae*
	Haemolytic uraemic syndrome (HUS)^b^	*Streptococcus pneumoniae*
	Neurological symptoms (e.g., encephalitis)^b^	*Mycoplasma pneumoniae*

MIRM, *M. pneumoniae*–induced rash and mucositis

^a^According to frequency

^b^Rare (< 1%)

^c^The prevalence of bacteraemia is increased in patients with local complications of CAP

^d^New definition to differentiate an association with infections (MIRM or reactive infectious mucocutaneus eruption [RIME]) from medications (Stevens-Johnson syndrome [SJS] or toxic epidermal necrolysis [TEN]) [19]

### *Mycoplasma pneumoniae*

The lack of a cell wall makes *M. pneumoniae* naturally resistant to first-line empirical beta-lactam antibiotics and a non-response to beta-lactam antibiotics is a reliable diagnostic indicator of *M. pneumoniae* infection [18]. Other features that may aid physicians in identifying patients at high risk for *M. pneumoniae* CAP include age > 5 years, prolonged prodromal symptoms (> 6 days), extrapulmonary manifestations (predominantly skin involvement), family with respiratory symptoms, or CRP and PCT levels that are normal or only slightly elevated [18, 19].

Macrolides are the recommended first-line treatment for *M. pneumoniae* infection [4, 5]. However, it is unclear if macrolides are effective for CAP caused by *M. pneumoniae* [20]. Extensive global macrolide use has led to alarming rates of *M. pneumoniae* resistance [18, 20]. Efficacy data and targeted prescription of macrolides are needed to reduce this emergence of AMR. CAP due to *M. pneumoniae* can be mild and self-limiting [12, 18]. This observation supports the hypothesis of an immune-mediated pathogenesis. Therefore, watchful waiting is a possible option in the case of suspected *M. pneumoniae* CAP when following the patient closely and providing safety-netting advice. If antibiotic treatment is considered, pathogen detection by PCR should be sought beforehand (Fig. 3). A randomized controlled non-inferiority trial of placebo versus macrolide antibiotics for *M. pneumoniae* infection in children with CAP (MYTHIC study) will investigate the efficacy of macrolides for *M. pneumoniae* infection (www.mythic-study.ch/en).

## Conclusion

Timely and reliable identification of the underlying pathogen is critical for initiating effective and tailored antibiotic treatment of CAP. However, no single diagnostic test applied to non-pulmonary specimens is able to reliably determine aetiology in childhood CAP. In addition to reducing diagnostic uncertainty, there is an urgent need to reduce antibiotic overuse and antimicrobial resistance in children with CAP. Thus, improved diagnostic methods are needed to accurately diagnose bacterial CAP and assess the true effect of antibiotic treatment. A simple and pragmatic management algorithm for childhood CAP may aid physicians in providing optimal and safe care while helping to reduce the prescribing of antibiotics.

## Abbreviations

AMR: Antimicrobial resistance
ASC: Antibody-secreting cell
BTS: British Thoracic Society
CAP: Community-acquired pneumonia
CRP: C-reactive protein
ELISpot: Enzyme-linked immunospot
Hib: *Haemophilus influenzae* type b
IDSA: Infectious Diseases Society of America
IPD: Invasive pneumococcal disease
NPI: Non-pharmaceutical intervention
PCR: polymerase chain reaction
PCT: Procalcitonin
PCV: Pneumococcal conjugate vaccine
RCT: Randomized controlled trial
RSV: Respiratory syncytial virus
URT: Upper respiratory tract
